# Optimization of treatment planning workflow and tumor coverage during daily adaptive magnetic resonance image guided radiation therapy (MR-IGRT) of pancreatic cancer

**DOI:** 10.1186/s13014-018-1000-7

**Published:** 2018-03-24

**Authors:** Sven Olberg, Olga Green, Bin Cai, Deshan Yang, Vivian Rodriguez, Hao Zhang, Jin Sung Kim, Parag J. Parikh, Sasa Mutic, Justin C. Park

**Affiliations:** 1 Department of Radiation Oncology, Washington University School of Medicine, St. Louis, MO 63110 USA; 2 Department of Radiation Oncology, Yonsei Cancer Center, Yonsei University College of Medicine, Seoul, South Korea

**Keywords:** Adaptive radiation therapy, MR-IGRT, Treatment planning, ViewRay™, Pancreatic cancer

## Abstract

**Background:**

To simplify the adaptive treatment planning workflow while achieving the optimal tumor-dose coverage in pancreatic cancer patients undergoing daily adaptive magnetic resonance image guided radiation therapy (MR-IGRT).

**Methods:**

In daily adaptive MR-IGRT, the plan objective function constructed during simulation is used for plan re-optimization throughout the course of treatment. In this study, we have constructed the initial objective functions using two methods for 16 pancreatic cancer patients treated with the ViewRay™ MR-IGRT system: 1) the conventional method that handles the stomach, duodenum, small bowel, and large bowel as separate organs at risk (OARs) and 2) the OAR grouping method. Using OAR grouping, a combined OAR structure that encompasses the portions of these four primary OARs within 3 cm of the planning target volume (PTV) is created. OAR grouping simulation plans were optimized such that the target coverage was comparable to the clinical simulation plan constructed in the conventional manner. In both cases, the initial objective function was then applied to each successive treatment fraction and the plan was re-optimized based on the patient’s daily anatomy. OAR grouping plans were compared to conventional plans at each fraction in terms of coverage of the PTV and the optimized PTV (PTV OPT), which is the result of the subtraction of overlapping OAR volumes with an additional margin from the PTV.

**Results:**

Plan performance was enhanced across a majority of fractions using OAR grouping. The percentage of the volume of the PTV covered by 95% of the prescribed dose (D_95_) was improved by an average of 3.87 ± 4.29% while D_95_ coverage of the PTV OPT increased by 3.98 ± 4.97%. Finally, D_100_ coverage of the PTV demonstrated an average increase of 6.47 ± 7.16% and a maximum improvement of 20.19%.

**Conclusions:**

In this study, our proposed OAR grouping plans generally outperformed conventional plans, especially when the conventional simulation plan favored or disregarded an OAR through the assignment of distinct weighting parameters relative to the other critical structures. OAR grouping simplifies the MR-IGRT adaptive treatment planning workflow at simulation while demonstrating improved coverage compared to delivered pancreatic cancer treatment plans in daily adaptive radiation therapy.

## Background

Magnetic resonance image guided radiation therapy (MR-IGRT) systems (MRIdian System; ViewRay™ Inc., Oakwood Village, OH) have been successfully implemented at a number of institutions in recent years and used to treat a growing group of patients that benefit from the advantages offered by Magnetic Resonance Imaging (MRI) compared to other conventional image guiding modalities (e.g. CBCT, x-ray radiography) [1, 2]. Anatomical variations from simulation to treatment as well as fraction to fraction represent a challenge in the delivery of radiation therapy, as changes in the size and position of target and critical structures can affect dose delivery in a clinically significant way [3–12]. The improved visualization of soft tissues gained from an MRI system compared to traditional cone-beam CT (CBCT) systems allows for the daily management of these inter-fraction anatomical variations, especially in areas of extensive soft tissue like the abdomen [1, 13]. It is this improved soft tissue visualization that makes adaptive radiation therapy (ART) an attractive application of the MR-IGRT system [1, 14, 15]. The implementation of ART does come at the cost of an increased time investment for plan re-optimization, however, which reflects the time consuming nature of inverse treatment planning in radiation therapy [1, 16].

Inverse planning involves the assignment of weighting parameters to target and critical structures that control the balance between delivering the prescribed dose to the target and protecting healthy tissues [17–23]. The selection of these parameters is recognized as a challenging undertaking that involves a “guessing game” of repeated trial and error in a “human iteration loop.” [22–26] This process becomes more complex and time consuming as the number of important structures and associated parameters involved in a plan grows, highlighting the need for a simpler treatment planning workflow [25, 27].

In pancreatic cancer cases specifically, a group of four structures – the stomach, duodenum, small bowel, and large bowel – represent organs at risk (OARs) of particular importance [28, 29]. The present study proposes the use of a single OAR structure that combines these four primary OARs, restricting the number of associated weighting parameters by a factor of four and thereby simplifying the treatment planning process. The aim of the study is to simplify the daily adaptive treatment planning workflow in the treatment of pancreatic cancer using the ViewRay™ System while maintaining tumor coverage that is robust to inter-fraction anatomical variations. The conventional daily adaptive treatment planning workflow is described along with the OAR grouping method, and comparative dosimetric data for 16 pancreatic cancer patients treated with daily adaptive MR-IGRT is presented.

## Methods

Sixteen pancreatic cancer patients previously treated with daily adaptive MR-IGRT were used as test cases, representing 208 adapted fractions. The volumes of the planning target volume (PTV), the contours of which are held constant throughout treatment for each patient, ranged from 57.7 cm^3^ to 356.3 cm^3^ with an average of 160.5 cm^3^. Clinically delivered treatment plans were used as a baseline for comparison at each fraction. The OAR grouping method was compared to the baseline in three metrics [1]: Percentage of the PTV covered by 95% of the prescribed dose (D_95_) [2]; D_95_ coverage of the PTV OPT; and [3] Percentage of the PTV covered by 100% of the prescribed dose (D_100_).

### OAR grouping method

**Fig. 1:**
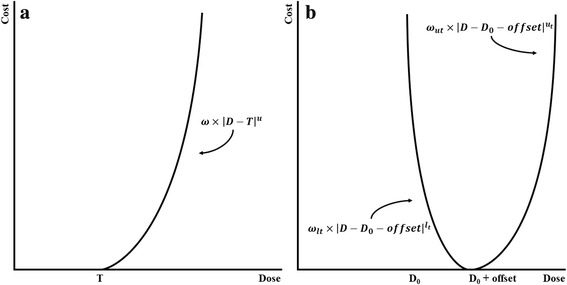
Representative cost functions plotted for an OAR (**a**) and the target structure (**b**), along with the associated weighting parameters

In the conventional treatment plan, each OAR is handled separately. Weighting parameters are assigned to each of these critical structures as well as the target as inputs to the objective function, which is the aggregate of the cost functions for individual structures involved in planning. Figure 1 illustrates the simple formulation of the cost function *f*(*D*| *θ*) used in this study for both OARs and the target, where *D* is the delivered voxel dose and θ is the set of weighting parameters assigned to a structure. For OARs (Fig. 1a), the cost increases for a given delivered dose *D* once a selected threshold *T* is exceeded. The shape of the curve is controlled using an importance factor ω and power *u*. Similarly, the cost for a dose *D* delivered to the target (Fig. 1b) increases as the dose deviates from the prescribed dose *D*_*0*_ plus a selected offset. The curves for doses above and below this threshold are shaped using importance and power parameters as discussed for the OAR case.

The creation of the initial objective function involves tuning each one of these parameters in order to achieve a plan of acceptable quality. In a pancreatic cancer treatment plan, each of the four primary OARs is subject to the same volumetric dose constraint (Table 1), so any variation in the assignment of weighting parameters is likely a reflection of the position of an OAR relative to the PTV. The OAR grouping method creates a single OAR structure by combining the portions of the stomach, duodenum, small bowel, and large bowel within 3 cm of the PTV. Using OAR grouping, the shared dose constraint is maintained, but the objective function is simplified by restricting the number of parameters used as inputs.

**Table 1:** Volumetric dose constraints observed in pancreatic cancer treatment plans. Each of the four primary OARs is subject to the same volumetric constraint of less than 0.5 cm^3^ receiving a maximum dose of 45 Gy

Structure	Volume Measure	Max Dose (Gy)
Stomach	< 0.5 cm^3^	45
Duodenum	< 0.5 cm^3^	45
Small Bowel	< 0.5 cm^3^	45
Large Bowel	< 0.5 cm^3^	45
Spinal Cord	= 0 cm^3^	40
Kidneys	< 50%	15

### Simplification of objective function

The value of the proposed method is derived from a simplification of the dose optimization function:

1 $$ {f}_{total}\left(D|\theta \right)=\sum \limits_{i=1}^k{f}_{ci}\left(D|{\theta}_i\right)+{f}_t\left(D|{\theta}_t\right), $$

where the cost *f* is a function of the delivered dose *D* and weighting parameters *θ*_*i*_ = {*ω*_*i*_, *u*_*i*_, *T*_*i*_} for all critical structures and *θ*_*t*_ = {*ω*_*lt*_, *ω*_*ut*_, *l*_*t*_, *u*_*t*_, *D*_0_, *offset*} for the target structure (Fig. 1). In the conventional pancreas treatment plan, the stomach, duodenum, small bowel, and large bowel each carry individual weighting parameters including upper importance *ω*, upper power *u*, and threshold *T*. Additionally, the target carries lower parameters for importance and power, *ω*_*l*_ and *l* respectively, as well as a prescribed dose *D*_0_ and corresponding offset. In this conventional case, the set of all weighting parameters for critical structures and the target*θ* = {*ω*_1_, *u*_1_, *T*_1_, …, *ω*_*k*_, *u*_*k*_, *T*_*k*_, *ω*_*lt*_, *ω*_*ut*_, *l*_*t*_, *u*_*t*_, *D*_0_, *offset*} is quite large. The OAR grouping method combines the four primary critical structures, and as a result [1] becomes

2 $$ {f}_{total}\left(D|\theta \right)={f}_c\left(D|{\theta}_c\right)+{f}_t\left(D|{\theta}_t\right)+\sum \limits_{j=1}^l{f}_{r- OAR}\left(D|{\theta}_j\right), $$

where *θ*_*c* + *t*_ = {*ω*_*c*_, *u*_*c*_, *T*_*c*_, *ω*_*lt*_, *ω*_*ut*_, *l*_*t*_, *u*_*t*_, *D*_0_, *offset*} represents the reduced set of weighing parameters for the four combined primary critical structures and the target and *θ*_*j*_ represents the weighting parameters of any remaining OARs (r-OAR) that may be included in the plan, such as the spinal cord or kidneys.

### Simulation

The conventional workflow for simulation has been previously described in detail [1, 15]. Briefly, patients undergo CT and MRI scans on the simulation day using the same setup device. Then, the MR images are sent to either the ViewRay™ treatment planning system or third-party software (e.g. Eclipse™) for the structure delineation. The corresponding structure sets, MR images, and CT scans are then combined and fused into the ViewRay™ treatment planning system for plan creation. Treatment isocenter, number of beam entries, and beam angles are defined by the planners such that the plan is physically deliverable with respect to the couch position. Finally, the prescribed dose and dose constraints on critical organs are used to guide the selection of weighting parameters input to the objective function for the IMRT plan optimization.

In this study, the conventional simulation plan was copied for each patient to keep the same beam entry and physical setup (treatment isocenter, couch position, etc.) in order to maintain as fair a comparison as possible. Using the OAR grouping method, portions of the stomach, duodenum, small bowel, and large bowel within 3 cm of the PTV were combined into a single OAR structure. This combined structure was then used in place of the individual critical structures in constructing the simulation objective function. The OAR grouping simulation plan was created and optimized such that the target coverage, OAR doses, and beam-on time were comparable to the conventional plan.

### Daily adaptive MR-IGRT

In daily adaptive MR-IGRT, the volumetric MRI of the patient is scanned before each treatment fraction. After critical structures are re-contoured, the plan is re-optimized based on the patient’s daily anatomy using the same objective function constructed in the pretreatment simulation plan, all while the patient remains on the couch. In the present study, the conventional plan at each treatment fraction was copied and modified using the OAR grouping method. For the purposes of comparison, all plans – both conventional and OAR grouping – were normalized to satisfy one of two scenarios, whichever came first: 1) the primary OAR receiving the greatest volumetric dose received a dose of 45 Gy to 0.5 cm^3^, or 2) the dose to the spinal cord or kidneys met the dose constraints as outlined in Table 1.

## Results

The percentage of fractions improved in each metric out of 208 total fractions for all patients is presented in Table 2. Generally, the coverage is improved across a majority of fractions when OAR grouping is utilized over the conventional method. Greater than 70% of fractions showed improvement in PTV OPT coverage, while approximately 80% of all fractions demonstrated improved PTV coverage using OAR grouping.

**Table 2:** Percentage of fractions in which coverage was improved using OAR grouping. Total = 208 fractions

	PTV OPT D_95_	PTV D_95_	PTV D_100_
Fractions Improved (%)	73	78	84

**Fig. 2:**
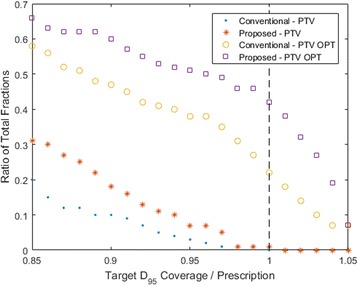
Cumulative histogram that demonstrates the ratio of total fractions receiving target coverage relative to the prescription of 95% target volume coverage by D_95_

In Fig. 2, PTV and PTV OPT D_95_ coverage relative to the prescription of 95% target volume coverage by D_95_ is plotted as a cumulative histogram. Figure 2 illustrates that PTV coverage fails to meet the prescription in nearly 100% of the adapted fractions due to the close proximity of surrounding OARs. The benefits of the proposed method can be more clearly understood when examining coverage of the PTV OPT, which is comprised of portions of the PTV not overlapped by OARs. In the conventional case, only 22% of all fractions exhibited PTV OPT coverage that met the prescription. When the OAR grouping method is utilized, that ratio increases to 42% of all fractions. It should be noted that the high ratio of under-covered fractions demonstrates the challenge of treating pancreatic cancer. In a majority of cases, the PTV is overlapped to some extent by surrounding OARs. In these cases, target coverage is often compromised in order to satisfy the dose constraints assigned to these critical structures.

**Fig. 3:**
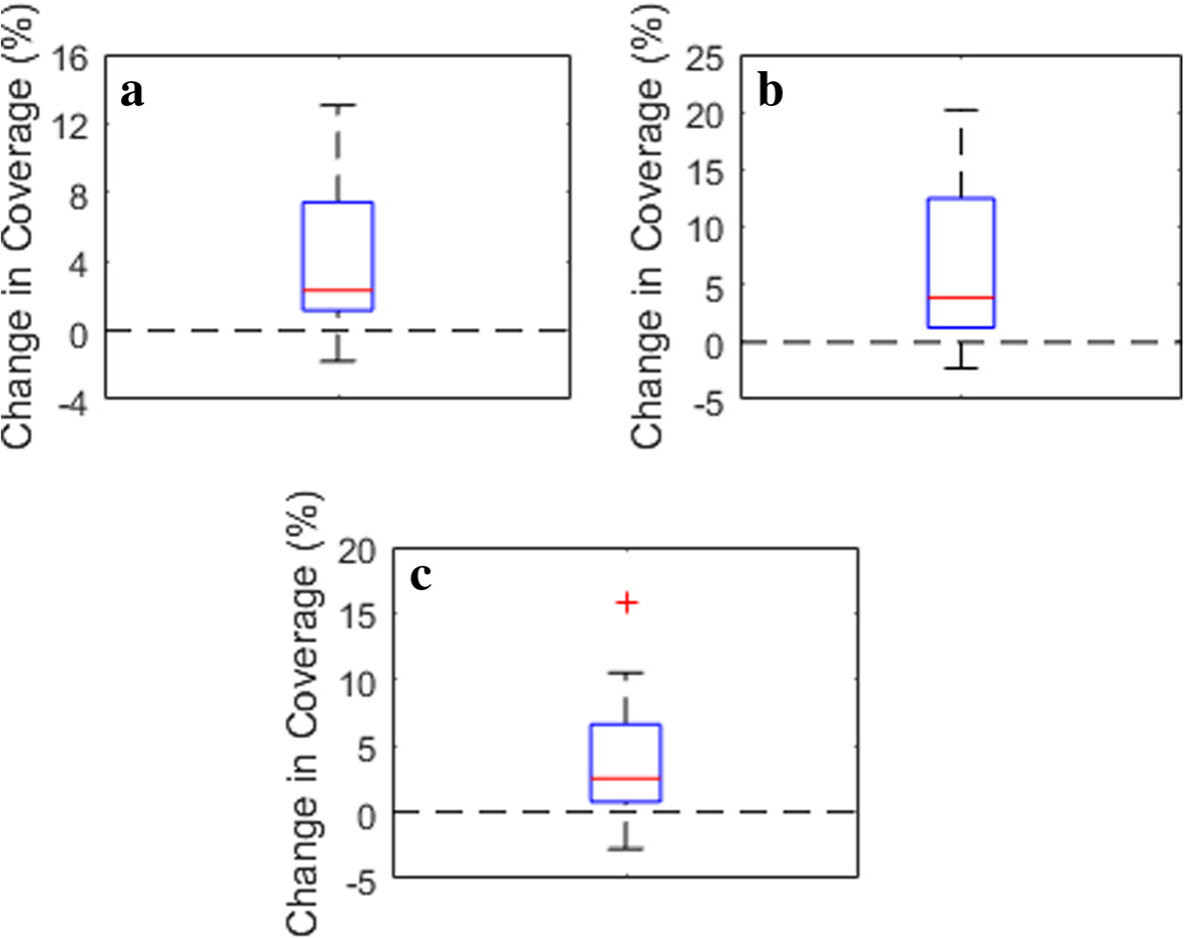
Average change from conventional to OAR grouping plans for each patient plotted for (**a**) PTV D_95_ coverage, (**b**) PTV D_100_ coverage, and (**c**) PTV OPT D_95_ coverage

Average coverage across a patient’s total adapted fractions is presented for each patient in Table 3 for both the conventional and OAR grouping plans. Similarly, Table 4 contains the average improvement of OAR grouping plans compared to conventional plans along with the minimum and maximum observed improvements across all patients. D_95_ coverage of the PTV and PTV OPT was improved by an average of approximately 4%, while PTV D_100_ coverage demonstrated an average improvement of greater than 6%. The relatively large standard deviations in each case are due to data points well above the mean, which can be observed for each metric in Fig. 3.

**Table 3:** Average coverage in each metric using the conventional and OAR grouping method over all adapted fractions for each patient

Adapted Fractions	Method	Average Coverage (%)		
		PTV OPT	PTV	
		D_95_	D_95_	D_100_
*Patient 1*				
14	Conventional	94.00	77.36	66.47
	Proposed	95.78	79.64	69.68
*Patient 2*				
13	Conventional	99.91	82.56	77.97
	Proposed	99.65	83.26	79.39
*Patient 3*				
13	Conventional	94.88	84.36	78.89
	Proposed	96.85	86.40	80.13
*Patient 4*				
15	Conventional	94.59	81.38	72.19
	Proposed	99.48	87.88	84.24
*Patient 5*				
14	Conventional	76.44	48.50	40.42
	Proposed	78.49	49.99	43.29
*Patient 6*				
15	Conventional	67.46	49.16	39.64
	Proposed	72.62	53.08	43.93
*Patient 7*				
14	Conventional	78.52	76.54	63.09
	Proposed	82.32	80.37	69.21
*Patient 8*				
10	Conventional	61.22	52.98	42.57
	Proposed	59.21	51.30	41.78
*Patient 9*				
13	Conventional	81.40	67.92	51.71
	Proposed	91.22	76.20	63.37
*Patient 10*				
14	Conventional	86.66	70.10	53.97
	Proposed	95.60	79.10	67.78
*Patient 11*				
14	Conventional	93.75	70.75	60.51
	Proposed	95.66	72.79	65.45
*Patient 12*				
11	Conventional	90.12	80.60	61.67
	Proposed	97.30	87.89	80.22
*Patient 13*				
6	Conventional	84.32	61.43	45.89
	Proposed	97.99	72.68	62.57
*Patient 14*				
13	Conventional	75.07	51.83	44.00
	Proposed	72.37	50.12	41.84
*Patient 15*				
14	Conventional	75.01	59.78	49.55
	Proposed	74.62	59.49	50.00
*Patient 16*				
15	Conventional	79.60	62.28	52.99
	Proposed	82.27	64.54	54.92

**Table 4:** Average, minimum, and maximum coverage differences between conventional and OAR grouping plans observed over all patients (*n* = 16)

		Average	Minimum	Maximum
PTV OPT	D_95_ (%)	3.98 ± 4.97	−2.78	15.87
PTV	D_95_ (%)	3.87 ± 4.29	−1.78	13.07
	D_100_ (%)	6.47 ± 7.16	−2.29	20.19

## Discussion

In the present study, the OAR grouping method was applied to pancreatic cancer cases with the aim of simplifying the daily adaptive MR-IGRT treatment planning workflow while maintaining target coverage that is robust to inter-fraction anatomical variations. In this way, the value of the proposed method is twofold. First, the simplification of the initial treatment planning process is easily understood. Reducing the number of OARs involved in planning restricts the set of weighting parameters that must be tuned iteratively to create an acceptable plan. Second and more important, the observed improvements in target coverage over a majority of fractions demonstrate the benefits of the method. The creation of the combined OAR structure makes the objective function created at simulation somewhat insensitive to significant changes in a patient’s anatomy from fraction to fraction. In the conventional plan, the weighting parameters selected at simulation may not accurately reflect the patient’s anatomy at a later treatment fraction. As a result, surrounding OARs may be overdosed and target coverage will suffer. In the proposed plan, the combined OAR structure lessens the impact of large differences in anatomy between simulation and treatment. Although the individual OARs may change position relative to the PTV, the position of the composite structure relative to the PTV changes less dramatically. As a result, the weighting parameters assigned at simulation more accurately reflect the present anatomy and target coverage is improved compared to the conventional case.

**Fig. 4:**
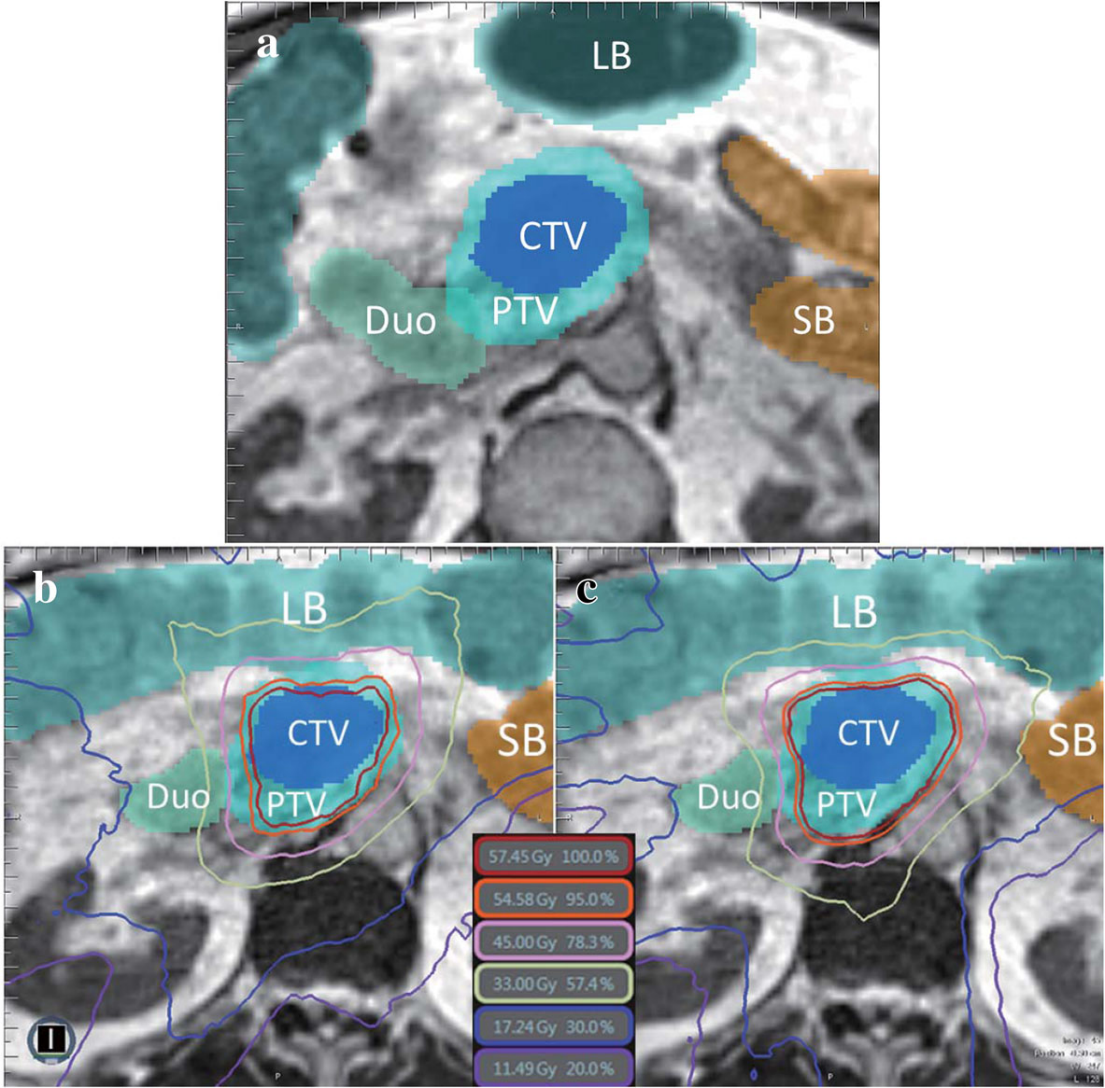
OARs and target structures in one slice at simulation (**a**) and treatment fraction 9 (**b**-**c**) for one patient. The large bowel demonstrates a large change in volume and proximity to the PTV from simulation to treatment. Isodose lines are displayed for the conventional plan (**b**) and the OAR grouping plan (**c**). The OAR grouping plan demonstrates improved D_100_ and D_95_ coverage over the PTV, as well as isodose lines that are moderately more conformal to the PTV compared to those in the conventional plan

**Fig. 5:**
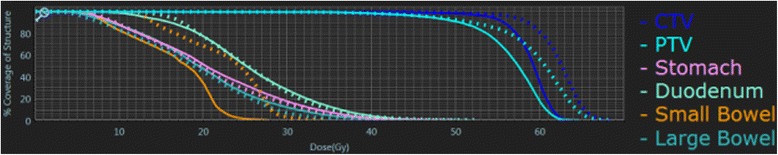
DVH for one patient comparing the conventional (solid lines) and OAR grouping (dashed lines) plans. Improved coverage of the PTV can be observed and OAR doses are generally comparable

A number of general trends were observed for individual patients as well as the cohort as a whole. Regarding the weighting selections made in conventional plans, two general situations are relevant. For plans in which an OAR was favored or disregarded by the conventional objective function through higher or lower weighting respectively, OAR grouping plans generally performed better in terms of coverage. Included as an illustrative example, Fig. 4 includes one slice from simulation (Fig. 4a) and the corresponding slice at treatment fraction 9 in the conventional and proposed plans for one patient (Fig. 4b-c). At simulation, the primary OARs in the conventional case were assigned weighting parameters based largely upon proximity to the PTV. Of the four primary OARs, the large bowel received the second lowest weighting due to the relatively small fraction of the structure located near the PTV, which is observable in Fig. 4a. The situation at fraction 9 is considerably different, as the large bowel now represents a significant volume in close proximity to the PTV. In the conventional case, the weighting assigned at simulation does not reflect the actual anatomy, and the large bowel is overdosed upon plan re-optimization as a result. Normalizing the delivered dose such that the large bowel receives the prescribed limit of 45 Gy to 0.5 cm^3^, D_100_ coverage of the PTV is only 14.29% in the conventional case (Fig. 4b). In contrast, by applying the OAR grouping method, the optimization function is made somewhat insensitive to these inter-fractional changes in anatomy and the resulting coverage demonstrates considerable improvement. As seen in Fig. 4c, the isodose lines in the OAR grouping plan are moderately more conformal to the PTV compared to those in the conventional plan, resulting in PTV D_100_ coverage of 74.93% without violating any OAR dose constraints. The dose-volume histogram (DVH) presented in Fig. 5 demonstrates improved PTV coverage and OAR doses that are generally comparable between the OAR grouping and conventional plans, save for the small bowel. The dose to the small bowel in this case, despite being higher in the OAR grouping plan, is still well below the volumetric dose limit assigned to the small bowel.

Now, the second scenario of note: for plans in which the OAR grouping method was approximated by the conventional objective function through the assignment of equal or similar weighting to each of the primary OARs, coverage for the conventional and proposed plans was generally comparable. It should be noted that the present study was limited in scope to cases in which in the volumetric dose constraints assigned to each of the four primary OARs were the same. Use of the OAR grouping method in cases where this condition is not maintained should be investigated further. However, it is anticipated that the OAR grouping method is valid as long as there does not exist any drastic difference between these critical structures.

## Conclusions

In this study, the OAR grouping method has been proposed as a means to simplify the daily adaptive treatment planning workflow and improve target coverage in adapted fractions. Characterizing the performance of OAR grouping plans reveals two scenarios of note. When the conventional simulation plan favored or disregarded an OAR through the assignment of distinct weighting parameters, OAR grouping plans generally demonstrated improved coverage compared to the conventional plan due to the decreased sensitivity of the OAR grouping objective function to inter-fraction anatomical variations. When the OAR grouping method was approximated by equal weighting in the conventional plan, coverage was generally comparable. In any case, the construction of the initial objective function at simulation is simplified by combining the four primary OARs in a pancreatic cancer case. This simplification comes along with an improvement in target coverage over a majority of fractions when comparing OAR grouping plans to conventional, clinically delivered plans.

## Abbreviations

ART: Adaptive radiation therapy
CBCT: Cone-beam CT
MRI: Magnetic Resonance Imaging
MR-IGRT: Magnetic resonance image guided radiation therapy
OAR: Organs at risk
PTV: Planning target volume
PTVOPT: Optimized planning target volume

## References

[1] Fischer-Valuck B, Henke L, Green O, et al. Two-and-a-half year clinical experience with the world’s first magnetic resonance image-guided radiation therapy system. Adv Radiat Oncol. 2017; 10.1016/j.adro.2017.05.006. 10.1016/j.adro.2017.05.006 PMC5605309 29114617

[2] Chen AM, Cao M, Hsu S (2017). Magnetic resonance imaging guided reirradiation of recurrent and second primary head and neck cancer. Adv Radiat Oncol.

[3] Evans PM (2008). Anatomical imaging for radiotherapy. Phys Med Biol.

[4] McDermott LN, Wendling M, Sonke JJ, van Herk M, Mijnheer BJ (2006). Anatomy changes in radiotherapy detected using portal imaging. Radiother Oncol.

[5] van Herk M (2004). Errors and margins in radiotherapy. Semin Radiat Oncol.

[6] Hector CL, Webb S, Evans PM (2000). The dosimetric consequences of inter-fractional patient movement on conventional and intensity-modulated breast radiotherapy treatments. Radiother Oncol.

[7] Schwartz DL, Garden AS, Thomas J (2012). Adaptive radiotherapy for head-and-neck cancer: initial clinical outcomes from a prospective trial. Int J Radiat Oncol Biol Phys.

[8] Ahunbay EE, Peng C, Chen GP (2008). An on-line replanning scheme for interfractional variations. Med Phys.

[9] Barker JL, Garden AS, Ang KK (2004). Quantification of volumetric and geometric changes occurring during fractionated radiotherapy for head-and-neck cancer using an integrated CT/linear accelerator system. Int J Radiat Oncol Biol Phys.

[10] Spoelstra FOB, Pantarotto JR, van Sörnsen de Koste JR, Slotman BJ, Senan S (2009). Role of adaptive radiotherapy during concomitant chemoradiotherapy for lung cancer: analysis of data from a prospective clinical trial. Int J Radiat Oncol Biol Phys.

[11] Surucu M, Shah KK, Roeske JC, Choi M, Small W, Emami B (2017). Adaptive radiotherapy for head and neck cancer: implications for clinical and dosimetry outcomes. Technol Cancer Res Treat.

[12] Nakamura M, Shibuya K, Nakamura A (2012). Interfractional dose variations in intensity-modulated radiotherapy with breath-hold for pancreatic cancer. Int J Radiat Oncol Biol Phys.

[13] Song WY, Kamath S, Ozawa S (2008). A dose comparison study between XVI® and OBI® CBCT systems. Med Phys.

[14] Mutic S, Dempsey JF (2014). The ViewRay system: magnetic resonance-guided and controlled radiotherapy. Semin Radiat Oncol.

[15] Wooten HO, Green O, Yang M (2015). Quality of intensity modulated radiation therapy treatment plans using a ^60^Co magnetic resonance image guidance radiation therapy system. Int J Radiat Oncol Biol Phys.

[16] Craft DL, Hong TS, Shih HA, Bortfeld TR (2102). Improved planning time and plan quality through multicriteria optimization for intensity-modulated radiotherapy. Int J Radiat Oncol Biol Phys.

[17] Barth NH (1990). An inverse problem in radiation therapy. Int J Radiat Oncol Biol Phys.

[18] Goitein M (1990). The inverse problem. Int J Radiat Oncol Biol Phys.

[19] Xing L, Chen GTY (1996). Iterative methods for inverse treatment planning. Phys Med Biol.

[20] Oelfke U, Bortfeld T (2001). Inverse planning for photon and proton beams. Med Dosim.

[21] Hamacher HW, Küfer KH (2002). Inverse radiation therapy planning – a multiple objective optimization approach. Discret Appl Math.

[22] Orton CG, Bortfeld TR, Niermierko A, Unkelback J (2008). The role of medical physicists and the AAPM in the development of treatment planning and optimization. Med Phys.

[23] Liu H, Dong P, Xing L. Using measurable dosimetric quantities to characterize the inter-structural tradeoff in inverse planning. Phys Med Biol. 2017; 10.1088/1361-6560/aa6fcb. 10.1088/1361-6560/aa6fcb 28447959

[24] Ezzell GA, Galvin JM, Low D (2003). Guidance document on delivery, treatment planning, and clinical implementation of IMRT: report of the IMRT subcommittee of the AAPM radiation therapy committee. Med Phys.

[25] Xing L, Li JG, Donaldson S, Le QT, Boyer AL (1999). Optimization of importance factors in inverse planning. Phys Med Biol.

[26] Bortfeld T (2006). IMRT: a review and preview. Phys Med Biol.

[27] Wu Q, Djajaputra D, Wu Y, Zhou J, Liu HH, Mohan R (2003). Intensity-modulated radiotherapy optimization with gEUD-guided dose-volume objectives. Phys Med Biol.

[28] Prior P, Chen X, Botros M (2016). MRI-based IMRT planning for MR-linac: comparison between CT- and MRI-based plans for pancreatic and prostate cancers. Phys Med Biol.

[29] Heerkens HD, Hall WA, Li XA (2017). Recommendations for MRI-based contouring of gross tumor volume and organs at risk for radiation therapy of pancreatic cancer. Pract Radiat Oncol.

